# Teaching others rule-use improves executive function and prefrontal activations in young children

**DOI:** 10.3389/fpsyg.2015.00894

**Published:** 2015-06-30

**Authors:** Yusuke Moriguchi, Yoko Sakata, Mikako Ishibashi, Yusuke Ishikawa

**Affiliations:** ^1^Department of School Education, Joetsu University of Education, Joetsu, Japan; ^2^Precursory Research for Embryonic Science and Technology, Japan Science and Technology Agency, Kawaguchi, Japan; ^3^Department of Psychology, Aichi Shukutoku University, Nagakute, Japan

**Keywords:** executive function, prefrontal cortex, teaching, pretend play

## Abstract

Intervention of executive function during early childhood is an important research topic. This study examined the effect of a child-friendly intervention program, where children interacted with a doll or a puppet. Children were presented with cognitive shifting tasks before and after an intervention. In the intervention, children interacted with a doll or a puppet, and taught rules of the cognitive shifting tasks to the object. As the results, 3- to 5-year-old children significantly improved the performances and strengthened activations in the lateral prefrontal regions as measured by near-infrared spectroscopy. The results suggest that interaction with a doll or a puppet may have a significant impact on the development of executive function.

## Introduction

Executive function refers to a self-regulatory process that controls cognition, emotion, and actions. Executive function shows dramatic changes during preschool years, subserved by the maturation of the prefrontal cortex (Zelazo et al., 1996; Carlson and Moses, 2001; Bunge and Zelazo, 2006; Moriguchi et al., 2007; Moriguchi and Hiraki, 2013b). Given that executive function during childhood has long-term effects and predicts socio-economic status and health status during adulthood (Casey et al., 2011; Moffitt et al., 2011), methods for intervention and training of executive function during early childhood is an important research topic (Rueda et al., 2005; Lillard and Else-Quest, 2006; Diamond et al., 2007; Diamond and Lee, 2011; Hostinar et al., 2012).

There are several effective approaches to improving executive function in young children, such as computer-based interventions, school curriculums, and reflection programs (Rueda et al., 2005; Lillard and Else-Quest, 2006; Diamond et al., 2007; Thorell et al., 2009; Moriguchi, 2012; Espinet et al., 2013). Recently, it has been proposed that reflective processing may be important for the development and training of executive function (Zelazo et al., 2003; Zelazo, 2004). According to this theory, reflective processing may help to formulate, select, and hold the structures or rules of a given task, which may lead to improvement of self-regulated cognitive processes. Indeed, reflection training improved children’s performances on executive function tasks and its neural correlates (N2 components) measured by event-related potential (Espinet et al., 2013). When children failed to perform the executive function tasks in these studies, they were given corrective feedback and taught to reflect on the rules and structures of the task.

Although such training may be effective for improving executive function, there have been some problems with previous studies. First, most of the programs have been teacher- or trainer-dependent. In school curriculums, the teacher training required several weeks to complete (Diamond and Lee, 2011). In computer-based training and reflective training, children were basically passive and received feedback from either a trainer or a computer (Thorell et al., 2009; Espinet et al., 2013). During the training, children had to sit in a chair for a long time while they passively received the programs. This style of training itself requires some extent of discipline and self-regulation on the part of the children. Thus, we believe that more child-friendly training should be provided. In addition, most of the previous studies have relied on behavioral measures to assess the effects of the intervention. Given that executive function is subserved by activations in the prefrontal cortex (Miller and Cohen, 2001; Moriguchi and Hiraki, 2014), an assessment of the neuroimaging method is also required.

Here, we proposed an intervention program that included children’s social interaction with a personified object (Moriguchi, 2014). Children enjoy interacting, talking, and playing with these objects, which is referred to as imaginary companion (IC) play (Bouldin and Pratt, 1999; Taylor et al., 2004; Fernyhough et al., 2007; Moriguchi and Shinohara, 2012; Motoshima et al., 2014). It has been shown that nearly half of young children enjoy interacting with an IC. Such play is observed not only in Western cultures, but also in Asian countries (Bouldin and Pratt, 1999; Taylor et al., 2004; Fernyhough et al., 2007; Moriguchi and Shinohara, 2012). Traditionally, when children interact with an invisible friend, it is treated as an instance of IC play (Svendsen, 1934). However, recently personified objects such as puppets and dolls have also been included (Taylor, 1999).

The relationship between IC play and executive function is still under debate. Theoretically, engaging pretend play may promote self-regulatory behaviors (but see also, Lillard and Else-Quest, 2006). Vygotsky (1962, 1967) proposed that children improve self-regulation through the interpersonal interaction, tools that aid attention and memory, and private speech as well as play. Specifically, rules included in imaginative play would lead children to act against immediate impulse. Carlson and Zelazo (2008) suggest that symbolic thought such as pretend play induce increasing level of reflection about symbol-referent relationship, which may correspond to the development of complex cognitive control processes (i.e., executive function). Nevertheless, there were mixed empirical data regarding the relationship between pretend play and executive function (Lillard et al., 2013). Moreover, the relationship between IC play and executive function was much more unclear, showing that some studies showed an advantage of IC play for preschool children’s executive skills, and other showed the disadvantage of IC play (Carlson and White, 2013).

The mixed results may be due to that previous studies have examined the overall relationship between IC play and executive function. Thus, the present study examined specific relation between play with a personified object and executive function. It has been shown that the relationship between a child and a personified object is more vertical than horizontal, and children usually take a parent-like role by providing guidance, didactic instruction and teaching to the IC (Gleason et al., 2000; Gleason, 2002). Thus, we focused on children’s teaching to personified objects.

To teach someone, we have to represent, hold, and reflect the content of what is being taught. Recent studies have shown that young children are willing to teach and that they sometimes enforce social rules with puppets as well as people (Tomasello, 2009; Rossano et al., 2011; Vaish et al., 2011). Efficient teaching may include reflective processing and flexible cognitive control mechanisms, as in those which comprise executive function (Strauss and Ziv, 2012). Indeed, there is evidence that children’s teaching skills are significantly correlated with executive function (Davis-Unger and Carlson, 2008). Thus, teaching may be a useful procedure for improving executive skills in young children.

In the present study, using behavioral and neuroimaging techniques, we examined whether children’s executive skills can be improved by training children to teach personified objects (i.e., doll, puppet). The personified objects may be more useful than another child because the object did not interfere with children’s teaching. Children were given the Dimensional Change Card Sort (DCCS) task, which is a widely used measure of developing executive function during preschool years (Zelazo et al., 1996; Moriguchi et al., 2010). We selected the DCCS task because previous studies have shown that the task was sensitive to index the development of executive function (Zelazo, 2006). In this task, children are asked to sort cards that have two dimensions, such as color and shape (e.g., red stars, blue cars). During the prestwich phase, children are asked to sort cards according to one dimension (e.g., color). During the postswitch phase, children are asked to sort the cards according to the other dimension (e.g., shape). Most 3-year-olds correctly perform the prestwich phase, but show difficulty with the postswitch phase. Zelazo et al. (2003) proposed that children have to reflect on conflicting rule representations and formulate higher-order rule systems that resolve the conflict on the DCCS tasks (but see also, Kirkham et al., 2003; Kloo and Perner, 2005). Importantly, reflection training improved children’s performances on the DCCS tasks and the N2 components (Espinet et al., 2013). Given that, if teaching facilitates reflective processing, children who teach the rule of the DCCS tasks would improve the performances on the DCCS tasks.

In this study, children were given the DCCS task during the first session and second session, and an intervention was given between the sessions. During the intervention, children interacted and talked with a personified object, and taught the rule of the DCCS task to it. In Study 1, we conducted a behavioral experiment. In Study 2, we measured the activation of the lateral prefrontal regions using near-infrared spectroscopy (NIRS). The differences in prefrontal activations between the first and second sessions were then compared.

## Study 1

### Materials and Methods

#### Participants

Thirty-two preschool Japanese children [aged 43.0 ± 3.0 months (mean ± SD)] participated in this study. Eight of the children performed the DCCS tasks perfectly in the first session, and were excluded from the analyses. Thus, 24 children [aged 43.0 ± 2.8 months (mean ± SD), 11 boys and 13 girls] comprised the final sample of the study. There were two experimental groups: a doll group and a control group. Children were randomly assigned to the two experimental groups. There were no significant age or sex differences between the groups (*t* < 1.0). The ages and sex of the participants were not different across groups.

Parents provided written informed consent for the children and were informed verbally about the purpose of the study. The study design was approved by the local ethics committee.

#### Materials

Laminated cards were used as stimuli. The stimuli had two dimensions: shape and color. The task included target cards and test cards; the target cards matched test cards in one dimension, but not in the other (e.g., target card: a yellow car and a green flower; test card: green cars and yellow flowers). There were two target cards and 12 test cards. We used the same stimuli across the first session, an intervention, and the second session. In the doll group, an experimenter’s doll was used.

#### Procedure

Children in both groups participated in the first session, an intervention, and the second session. During the first session, a child was given the DCCS task. The child was instructed to sort the cards according to one dimension (e.g., in the shape game, “This is a shape game. All the cars go here and all the flowers go there”). In this prestwich phase, the child was given 12 trials, and at the beginning of each trial, the experimenter told the child the rule of the game, randomly selected a sorting card, and asked him/her to sort the cards. The child was required to place the card on one of the two trays. The child was given feedback on every trial (“Yes”/“No”). When they had completed the first phase of the task, the child was asked to stop playing the game and told to switch to a new game. If the child sorted the cards according to the shape dimension in the first phase, he/she was asked to sort cards according to the color dimension (e.g., “The new game is a color game. The color game is different from the shape game. In the color game, all the yellow ones go here and all the green ones there.”). The child was then given 12 trials that were identical to those in the prestwich phase except for the dimension (e.g., color). In the postswitch phase, the child was not given feedback as to whether he/she sorted the cards correctly. The order of dimensions was counterbalanced between children as to whether they received color or shape first.

At the beginning of the intervention, a child in the doll group would be given a practice phase. In the practice phase, the experimenter taught the child rules of the tasks as in the prestwich and the postswitch phases in the first session. Then, the child was asked to sort the cards according to the first and the second rule for 12 trials respectively. The feedback was given to the child when he/she made errors. After the practice phase, a child in the doll group was asked to interact with the doll. The experimenter told the child that the doll was a friend of the experimenter, but that the doll did not know the rules of the game (the DCCS tasks). Then, the child was asked to teach the doll the rules of the prestwich and the postswitch phases on the DCCS tasks (e.g., during the prestwich phases of the shape game, “Please teach her how to sort the cards in the shape game” During the postswitch phases of the color game, “OK, now we will change the rule of the game. Please teach her how to sort the cards in the color game”). All of the children were willing to teach the rules to the doll. When a child taught the wrong rule in the second phase, the experimenter informed the child about the error and corrected the error.

The procedure for the control group was identical to that of the doll group, except that a child in the control group was not introduced to a doll. Rather, a child in the control group was given a practice phase, where the experimenter taught the child rules of the tasks twice. The amount of time (on average 2 min) provided for the intervention in the control group was matched to that provided for the doll group.

The second session proceeded exactly the same way as in the first session. The entire experiment was conducted on the same day.

### Results and Discussion

**FIGURE 1:**
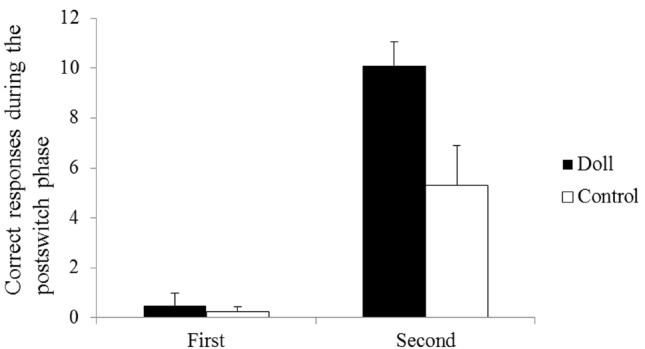
**Results of Study 1.** The number of correct responses during the postswitch phase in the first and second sessions. Error bars indicate SE.

The measurements of the performances were compared across groups (Figure 1). In the first session, children in both the doll group and control group showed difficulty with the postswitch phases. In the second session, however, the children in the doll group easily performed the postswitch phase. The number of correct responses was analyzed using a group (doll vs. control) × session (first vs. second) × phase (prestwich vs. postswitch) three-way mixed ANOVA. There was a significant main effect of session [*F* (1, 22) = 9.617, *p* < 0.01, ${\text{η}}_{p}^{2}$ = 0.30] and phase [*F* (1, 22) = 62.723, *p* < 0.001, ${\text{η}}_{p}^{2}$ = 0.74], but no significant main effect of group [*F* (1, 22) = 2.312, *p* > 0.10, ${\text{η}}_{p}^{2}$ = 0.10]. Importantly, we found a significant group × session × phase three-way interaction [*F* (1, 22) = 4.523, *p* < 0.05, ${\text{η}}_{p}^{2}$ = 0.17].

The three-way interaction was followed up with 2 (group) × 2 (session) ANOVAs for each phase. For prestwich phase there were no significant interactions between group and session, [*F* (1, 22) = 0.410, *p* > 0.10, ${\text{η}}_{p}^{2}$ = 0.02]. On the other hand, for postswitch phase there was a significant interaction between group and session, [*F* (1, 22) = 5.827, *p* < 0.02, ${\text{η}}_{p}^{2}$ = 0.21]. *Post hoc* analyses using the Bonferroni method revealed that a simple-simple main effect for the group during the second session in the postswitch phase was significant (*p* < 0.05), showing children in the doll group performed significantly better than those in the control group.

The results revealed that children in the doll group significantly improved their performances in the postswitch phase through their interactions with the doll over children in the control group. Study 2 aimed to extend the findings and examined whether the same training effects can be observed at the neural level. If we found the training effects at the neural level, we may use the brain activations as neural markers of the training effects. Moreover, individual differences in the training effect were unclear in Study 1. It was possible that children who have an IC may gain a greater benefit from interacting with personified objects than those without an IC, because children with IC had experience of interacting with a puppet or a doll on a daily basis, and therefore they would easily interact with the personified objects in an experimental setting. It has been shown that children had significant activation in the lateral prefrontal regions during the DCCS task and that such activations were related to the children’s performances on the DCCS (Moriguchi and Hiraki, 2009, 2011). The activations in the prefrontal regions were observed during the prestwich and the postswitch phases. Moriguchi and Hiraki (2013a) suggested that the prefrontal activations may reflect the representations of the higher-order rules as suggested by Zelazo et al. (2003). If this were the case, the reflective training would improve the activations in the prefrontal regions. In Study 2, we examined activations in the lateral prefrontal regions during the DCCS tasks before and after the intervention. In addition, we conducted an IC interview to assess whether children had ICs. We recruited 5-year-old children for Study 2 because the intervention research took a longer time to conduct than average neuroimaging studies, and our pilot study showed that younger children showed difficulty with the length of the experiment.

## Study 2

### Materials and Methods

#### Participants

Nineteen preschool Japanese children [aged 61.2 ± 7.6 months (mean ± SD)] participated in this study, but four children were excluded from the analyses because we failed to measure any neural activations in their target regions. Thus, 15 children [aged 62.1 ± 8.4 months (mean ± SD), 7 boys and 8 girls] comprised the final sample in the study.

Parents provided written informed consent for the children and were informed verbally about the purpose of the study. The study design was approved by the local ethics committee.

#### Behavioral Experiment

**FIGURE 2:**
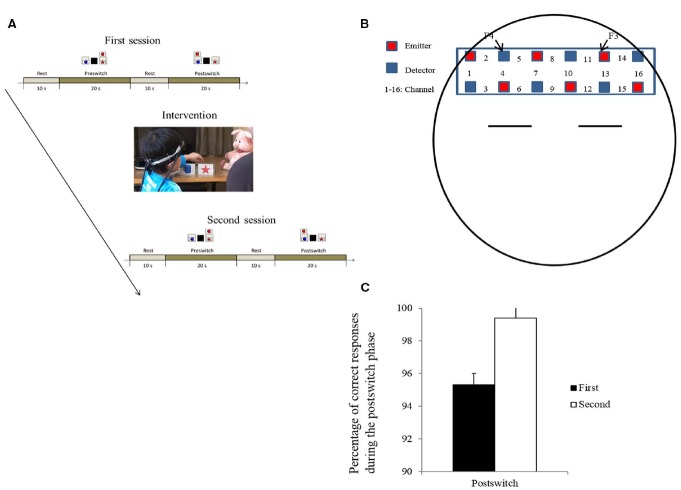
**Experimental settings and results of Study 2. (A)** Experimental procedures. **(B)** The NIRS probe was attached to the bilateral prefrontal areas. Each channel consisted of one emitter optode and one detector optode. The region of interest was located near F3/4, which roughly corresponds to ch 11, 13, 14, and ch 2, 4, 5, respectively. **(C)** Behavioral results. Error bars indicate SE.

The behavioral experiment was the same as in the doll group in Study 1, except for the following three points. First, a pig puppet was used instead of a doll (Figure 2A). Second, a child was given three DCCS tasks during both the first and second sessions. In each task, one session consisted of a rest phase (10 s), a prestwich phase (20 s), a second rest phase (10 s), and a postswitch phase (20 s). During the rest phases, children were asked to sit still. In Study 2, we used three pairs of target and test cards (e.g., a red star, a blue cup, red cups, and blue stars/an orange face, a purple flower, orange flowers, and purple faces). Third, there was no control condition in Study 2 for technical reasons. The NIRS signal is the product of the optical path length and the hemoglobin changes. Importantly, the optical path length would differ across children (Moriguchi and Hiraki, 2013b). Thus, the comparison of data between different groups was difficult.

#### Imaginary Companion Interview

The basic procedure of the interview followed the method used by Taylor et al. (1993). After playing with each child for several minutes, the experimenter asked the child about ICs (“I’m going to ask you some questions about your friends. Some friends are real, like the kids who go to your school and the ones with whom you play. And some friends are imaginary. They might be invisible, or they might be a puppet or doll. Do you have an imaginary friend, or have you ever had one?”). If the child answered “yes,” then the researcher asked the child questions about their imaginary friend (e.g., gender, age, physical appearance).

We also conducted the IC interview separately with the child’s parent. To avoid confusion, stories about an invisible friend and a personified object were told. It was explained that children of this age are often secretive about their ICs and that it is common for parents to be unaware of such friends. Mothers were then asked if they were aware of their children engaging in IC play either currently or at previous times. If they responded yes, the remainder of the IC interview was conducted. Children were considered to have an IC if the child or their parents indicated the presence of an IC, either currently or in the past. Mothers were also asked about the children’s reports of ICs to clarify whether the children had mistakenly identified a real friend. Such incorrect reports by children were not included as evidence of an IC.

#### NIRS Recordings and Analysis

Near-infrared spectroscopy measurements were performed during the first and second sessions. A multichannel NIRS unit operating at wavelengths of 770 and 840 nm (OEG-16; Spectratech Inc., Tokyo, Japan) was used to measure temporal changes in the concentrations of oxy-Hb and deoxy-Hb. The NIRS probes included 12 optodes, which constituted 16 channels. The probes were placed on the lateral prefrontal areas of each hemisphere. Each channel consisted of one emitter optode and one detector optode, which were located 3 cm apart. The temporal resolution at each channel was approximately 666 ms.

The region of interest (ROI) was located near F3/4 of the International 10/20 system, which corresponds to Brodmann areas (BA) 9/46, because previous studies have shown that these areas were activated during the DCCS (Morton et al., 2009). The spatial resolution of the NIRS is relatively low, and therefore, ch 11, 13, 14 and ch 2, 4, 5 were defined as corresponding to the left lateral and right prefrontal regions, respectively.

We set a low-pass filter (= 0.05 Hz) using fast Fourier transform (FFT) to exclude artifacts caused by any minor movements of the child. We also removed the motion artifact by the video recordings and our criterion of the NIRS data. Variations for each sample data were calculated by subtracting a previous data from a current data. Channels where a variation more than three standard deviations (SDs) was detected were excluded from further analysis. Approximately 5% of the data were excluded from the analyses.

From the NIRS parameters measured, the concentrations of oxy-Hb and deoxy-Hb were found to be sensitive to changes in regional cerebral blood flow, which provided a strong correlation with the blood oxygen level dependent (BOLD) signal in the prefrontal cortex (Sato et al., 2013). Thus, we analyzed changes in oxy-Hb and deoxy-Hb.

Changes in oxy-Hb and deoxy-Hb were analyzed from 5 to 20 s after the onset of the prestwich and postswitch phases, because of the time required to provide instructions concerning the rules (first 5 s in each phase; Moriguchi and Hiraki, 2009). The average changes in oxy-Hb during the prestwich and postswitch phases were calculated for all channels and in each subject.

We measured changes in oxygenated hemoglobin (oxy-Hb) and deoxygenated hemoglobin (deoxy-Hb) in the lateral prefrontal areas during the rest phases and task phases (prestwich and postswitch phases), and subtracted the changes during the rest phases from those during the task phases. As in previous NIRS studies, to reduce the signal-noise ratio, we aggregated ch 11, 13, 14 into the left lateral prefrontal area and ch 2, 4, 5 into the right lateral prefrontal area. The regions were located near F3/4 in the International 10/20 method, which correspond to the dorsolateral prefrontal regions (Okamoto et al., 2004; Figure 2B).

### Results and Discussion

At the behavioral level, the children’s performances improved after the intervention (Figure 2C). The mean percentage of correct responses was analyzed using a session (first vs. second) × phase (prestwich vs. postswitch) two-way repeated ANOVA. We found a marginally significant interaction between session and phase [*F* (1, 14) = 3.716, *p* < 0.08, ${\text{η}}_{p}^{2}$ = 0.21]. Thus, although there might be ceiling effects, the results of Study 2 partially replicated those of Study 1.

**FIGURE 3:**
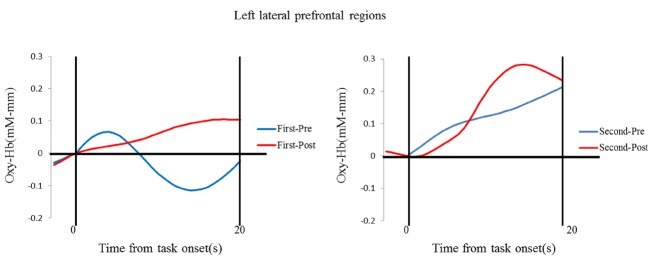
**Temporal change in the oxyhemoglobin concentration in the left lateral prefrontal areas.** Data for the group mean during the prestwich (blue line) and postswitch (red line) phases in the first session (left) and second session (right).

**FIGURE 4:**
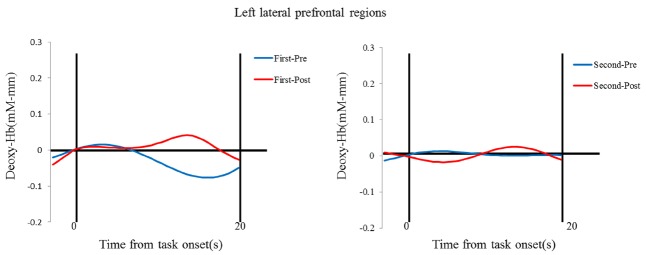
**Temporal change in the deoxyhemoglobin concentration in the left lateral prefrontal areas.** Data for the group mean during the prestwich (blue line) and postswitch (red line) phases in the first session (left) and second session (right).

The results of the NIRS recordings revealed that activations increased in the left prefrontal regions through teaching the puppet (Figures 3 and 4). The mean changes in oxy-Hb were analyzed using a laterality (right vs. left) × session (first vs. second) × phase (prestwich vs. postswitch) repeated-measures ANOVA. There were no significant main effects of laterality, session, or phase [*F* (1, 14) = 1.179, *p* > 0.10, ${\text{η}}_{p}^{2}$ = 0.01; *F* (1, 14) = 1.690, *p* > 0.10, ${\text{η}}_{p}^{2}$ = 0.02; *F* (1, 14) = 0.184, *p* > 0.10, ${\text{η}}_{p}^{2}$ = 0.01]. We found a significant interaction between laterality and session [*F* (1, 14) = 5.925, *p* < 0.02, ${\text{η}}_{p}^{2}$ = 0.05]. *Post hoc* analyses using the Bonferroni method revealed that a simple main effect for session in the left prefrontal region, but not in the right prefrontal region, was significant (*p* < 0.05), showing the significant stronger activations during the second sessions than those during the first sessions.

Finally, we examined individual differences in the increases in prefrontal activations. It is possible that children who have experience with an IC may gain a greater benefit from interacting with personified objects than those without an IC. Nine children were classified as the IC group, and the remaining six children were classified as the No Imaginary Companion (NIC) group. No significant differences were demonstrated between the ages of the two groups. In the IC group, one child had invisible friends and 8 had personified objects. One child in the IC group had only one IC, while the remaining children had two or more ICs. A child’s invisible friend consisted of people, with most of the children’s personified objects being stuffed animals (fox, penguins, bears, and so on). Eight children currently had ICs, and one child had stopped playing with their ICs prior to testing.

Given the results above, we focused on activations in the left prefrontal regions. We calculated the differences scores between the activations in the first session and the second session during the prestwich and postswitch phases. Moreover, we calculated the behavioral differences between the first and the second sessions during the prestwich and postswitch phases. Then, we compared the difference behavioral scores in the IC group to the NIC group. The difference behavioral scores were analyzed using a group (IC vs. NIC) × phase (prestwich vs. postswitch) mixed ANOVA. The analyses of behavioral scores revealed the significant main effects of phase [*F* (1, 13) = 6.335, *p* < 0.05, ${\text{η}}_{p}^{2}$ = 0.33], but no significant main effects of group and no significant interaction [*F* (1, 13) = 0.092, *p* > 0.10, ${\text{η}}_{p}^{2}$ = 0.01, *F* (1, 13) = 4.323, *p* > 0.05, ${\text{η}}_{p}^{2}$ = 0.25, respectively]. The difference behavioral scores were not significantly different across the IC and NIC group.

With regards to the difference neural scores, the comparison of data between different groups was difficult (Moriguchi and Hiraki, 2013b). Thus, we conducted one-sample *t*-tests to examine whether the difference neural scores in each group were different from 0. The analyses above showed no significant effect of phase, and therefore we aggregated the difference neural scores during the prestwich and the postswitch phases. The results showed that the difference neural scores were significantly different from 0 in IC group, but not in NIC group [*t* (8) = 2.827, *p* < 0.03, *d* = 0.93, *t* (5) = –0.441, *p* > 0.67, *d* = 0.18, respectively]. Thus, children with an IC showed the significant improvement at the neural level between the first and second sessions, but children without IC did not. Since the analyses did not directly compare the prefrontal activations in the IC group to those in the NIC groups, the results suggest that there might be some training effects in the IC group, but not in the NIC group.

## General Discussion

The present study provides the first behavioral and neuroimaging data demonstrating that interacting with a personified object may improve children’s executive function. Specifically, behavioral results revealed that teaching a doll was more effective in improving children’s performances on the DCCS tasks than only practicing the DCCS tasks (i.e., the control group). Moreover, such interaction increased the activations in the left prefrontal regions during the DCCS tasks in young children.

It has been shown that children’s performances on DCCS tasks improve between 3 and 5 years of age, and this development is subserved by activations in the lateral prefrontal regions (Zelazo et al., 1996, 2003; Moriguchi and Hiraki, 2009, 2011). Children who performed the DCCS tasks perfectly showed significant activations in the lateral prefrontal regions, whereas those who showed difficulty with the DCCS tasks failed to activate the prefrontal regions (Moriguchi and Hiraki, 2009). The results in the present study are basically consistent with the previous results, and extend the finding that intervention for executive function may increase activations in the prefrontal regions. Moreover, through the interaction with the puppet in Study 2, children increased activations in the left prefrontal regions, but not in the right prefrontal regions. Through the intervention, children in the second session may reflect and represent the task structure or rules in different ways from that in the first session in order to teach the puppet. As seen in the results, during the second session, children may have performed the DCCS tasks differently from the first session, which may be related to stronger activations in the left prefrontal cortex. Given the previous evidence that the left-lateralization may be related to verbal processing in a working memory study (Tsujii et al., 2009), children may use verbal strategies to perform the DCCS tasks. One might argue that children may improve the prefrontal activations in the second session because children received the three pairs of stimuli in the first sessions and the exposure may work as a training. However, previous studies have shown that intervention of EF is not so easy (Moriguchi, 2012). Thus, such exposure may not lead to the improvements in the prefrontal activations.

The results showed that in developing executive function, interaction with a personified object may act as a more efficient method of training than merely practicing. However, the present study did not address whether our methodology may be more effective than other intervention programs. Moreover, it is possible that interaction with an experiment or another child may enhance children’s performances, because we believe that interaction with a puppet or a doll would enhance reflective processing, and the same effects can be observed in interaction with a human. The advantage of our method is that it is child-friendly and ecologically valid. Children were willing to interact, talk, and teach the rules of the tasks to the puppets, and seemed to enjoy participating in the intervention program.

Thus, our method may be efficient for children who show difficulty with receiving traditional education programs, or with participating in computer-based training. Indeed, it has been shown that children with developmental disorders, such as attention deficit hyperactivity disorder or autism, have difficulty with executive function tasks (Hill, 2004; Mulas et al., 2006; Johnson, 2012). Some of the children may experience difficulty interacting with people, or with sitting in a chair for the computer-based training. Such children may benefit from intervention using doll or puppet play.

The present results contribute to our understanding of children’s IC play. It has been shown that nearly half of pre-school children enjoy interacting with pretend friends. There are several theories on the developmental effects of IC play. For example, children may be training their socio-cognitive skills through interaction with an IC and by simulating an IC’s mental states (Harris, 2000). The other role of an IC may be to compensate for loneliness or a lack of relationships in the real world (Singer and Singer, 1990). However, the relationship between executive function and IC play remains unclear (Carlson and White, 2013). The present study shows that interacting with a personified object may play an important role in improving executive function in young children. Moreover, we have shown that children with an IC received some benefits from this form of intervention. The results suggest that IC play can be an important form of play to develop executive function for children of this age.

Finally, it is necessary to consider the limitations of the present study. First, because of the technical limitations, only older children participated in Study 2. Therefore, an equivalent examination of neural activation in younger children is required. Second, we did not include transfer tasks. The DCCS tasks require reflective processing and teaching may improve reflective processing, which may lead to the improvement of the DCCS tasks. Determining whether reflection through teaching can be generalized to other tasks is an important next step in assessing the program. Third, there were no control conditions in study 2. The NIRS signal is the product of the optical path length and the hemoglobin changes, and the optical path length differs across participants and head positions (Zhao et al., 2002; Moriguchi and Hiraki, 2013b). Thus, the comparison of data between different children may be difficult. Nevertheless, we can include both the control and the training conditions within the same children. Forth, in Study 2, the number of children with and without IC was small, and how effective the training of executive function in each group was still unclear. Moreover, most of the children with IC (eight out of nine) were girls whereas most of the children without IC (four out of five) were boys. Thus, we did not determine whether the observed differences were due to sex differences or IC differences. The issue should be addressed in future studies. Moreover, in Study 1, it is possible that children in the doll condition received different types of experience (practice and teaching to a doll), which may lead to their superior performances. We had to assess the effect of different experiences on children’s performances. Finally, it is still unclear whether the effects observed in this study were specific to interaction with a personified object. Rather, it is possible that interaction with an experiment or another child may enhance children’s performances. Further studies are needed to assess whether other regions of the brain may also be involved in the improvement of executive skills.

### Conflict of Interest Statement

The authors declare that the research was conducted in the absence of any commercial or financial relationships that could be construed as a potential conflict of interest.

## References

[B1] Bouldin P. Pratt C. (1999). Characteristics of preschool and school-age children with imaginary companions. J. Genet. Psychol. 160, 397–410. 10.1080/00221329909595553 10584318

[B2] Bunge S. A. Zelazo P. D. (2006). A brain-based account of the development of rule use in childhood. Curr. Dir. Psychol. Sci. 15, 118–121. 10.1111/j.0963-7214.2006.00419.x

[B3] Carlson S. M. Moses L. J. (2001). Individual differences in inhibitory control and children’s theory of mind. Child Dev. 72, 1032–1053. 10.1111/1467-8624.00333 11480933

[B4] Carlson S. M. White R. E. (2013). “Executive function, pretend play, and imagination,” in The Oxford Handbook of the Development of Imagination, ed. Taylor M. (New York: Oxford University Press), 161–174.

[B5] Carlson S. M. Zelazo P. (2008). Symbolic thought. Encycl. Infant Early Child. Dev. 3, 288–297. 10.1016/b978-012370877-9.00158-4

[B6] Casey B. Somerville L. H. Gotlib I. H. Ayduk O. Franklin N. T. Askren M. K. (2011). Behavioral and neural correlates of delay of gratification 40 years later. Proc. Natl. Acad. Sci. U.S.A. 108, 14998–15003. 10.1073/pnas.1108561108 21876169 PMC3169162

[B7] Davis-Unger A. C. Carlson S. M. (2008). Children’s teaching skills: the role of theory of mind and executive function. Mind Brain Educ. 2, 128–135. 10.1111/j.1751-228X.2008.00043.x

[B8] Diamond A. Barnett W. S. Thomas J. Munro S. (2007). The early years—Preschool program improves cognitive control. Science 318, 1387–1388. 10.1126/science.1151148 18048670 PMC2174918

[B9] Diamond A. Lee K. (2011). Interventions shown to aid executive function development in children 4 to 12 years old. Science 333, 959–964. 10.1126/science.1204529 21852486 PMC3159917

[B10] Espinet S. D. Anderson J. E. Zelazo P. D. (2013). Reflection training improves executive function in preschool-age children: behavioral and neural effects. Dev. Cogn. Neurosci. 4, 3–15. 10.1016/j.dcn.2012.11.009 23280362 PMC6987836

[B11] Fernyhough C. Bland K. Meins E. Coltheart M. (2007). Imaginary companions and young children’s responses to ambiguous auditory stimuli: implications for typical and atypical development. J. Child Psychol. Psychiatry 48, 1094–1101. 10.1111/j.1469-7610.2007.01789.x 17995485

[B12] Gleason T. R. (2002). Social provisions of real and imaginary relationships in early childhood. Dev. Psychol. 38, 979–992. 10.1037/0012-1649.38.6.979 12428709

[B13] Gleason T. R. Sebanc A. M. Hartup W. W. (2000). Imaginary companions of preschool children. Dev. Psychol. 36, 419–428. 10.1037/0012-1649.36.4.419 10902694

[B14] Harris P. L. (2000). The Work of the Imagination. Oxford: Wiley-Blackwell.

[B15] Hill E. L. (2004). Executive dysfunction in autism. Trends Cogn. Sci. 8, 26–32. 10.1016/j.tics.2003.11.003 14697400

[B16] Hostinar C. E. Stellern S. A. Schaefer C. Carlson S. M. Gunnar M. R. (2012). Associations between early life adversity and executive function in children adopted internationally from orphanages. Proc. Natl. Acad. Sci. U.S.A. 109(Suppl. 2), 17208–17212. 10.1073/pnas.1121246109 23047689 PMC3477377

[B17] Johnson M. H. (2012). Executive function and developmental disorders: the flip side of the coin. Trends Cogn. Sci. 16, 454–457. 10.1016/j.tics.2012.07.001 22835639

[B18] Kirkham N. Z. Cruess L. Diamond A. (2003). Helping children apply their knowledge to their behavior on a dimension-switching task. Dev. Sci. 6, 449–467.

[B19] Kloo D. Perner J. (2005). Disentangling dimensions in the dimensional change card-sorting task. Dev. Sci. 8, 44–56. 15647066 10.1111/j.1467-7687.2005.00392.x

[B20] Lillard A. S. Else-Quest N. (2006). The early years: evaluating montessori. Science 313, 1893–1894. 10.1126/science.1132362 17008512

[B21] Lillard A. S. Lerner M. D. Hopkins E. J. Dore R. A. Smith E. D. Palmquist C. M. (2013). The impact of pretend play on children’s development: a review of the evidence. Psychol. Bull. 139, 1–34. 10.1037/a0029321 22905949

[B22] Miller E. K. Cohen J. D. (2001). An integrative theory of prefrontal cortex function. Annu. Rev. Neurosci. 24, 167–202. 10.1146/annurev.neuro.24.1.167 11283309

[B23] Moffitt T. E. Arseneault L. Belsky D. Dickson N. Hancox R. J. Harrington H. (2011). A gradient of childhood self-control predicts health, wealth, and public safety. Proc. Natl. Acad. Sci. U.S.A. 108, 2693–2698. 10.1073/pnas.1010076108 21262822 PMC3041102

[B24] Moriguchi Y. (2012). The effect of social observation on children’s inhibitory control. J. Exp. Child Psychol. 113, 248–258. 10.1016/j.jecp.2012.06.002 22781163

[B25] Moriguchi Y. (2014). The early development of executive function and its relation to social interaction: a brief review. Front. Psychol. 5:388. 10.3389/fpsyg.2014.00388 24808885 PMC4010730

[B26] Moriguchi Y. Hiraki K. (2009). Neural origin of cognitive shifting in young children. Proc. Natl. Acad. Sci. U.S.A. 106, 6017–6021. 10.1073/pnas.0809747106 19332783 PMC2667026

[B27] Moriguchi Y. Hiraki K. (2011). Longitudinal development of prefrontal function during early childhood. Dev. Cogn. Neurosci. 1, 153–162. 10.1016/j.dcn.2010.12.004 22436437 PMC6987577

[B28] Moriguchi Y. Hiraki K. (2013a). “Developmental relationship between executive function and the prefrontal cortex in young children,” in Prefrontal Cortex: Developmental Differences, Executive and Cognitive Functions and Role in Neurological Disorders, eds Collins R. O. Adams J. L. (New York: Nova Science Pub Inc.,), 155–174.

[B29] Moriguchi Y. Hiraki K. (2013b). Prefrontal cortex and executive function in young children: a review of NIRS studies. Front. Hum. Neurosci. 7:867. 10.3389/fnhum.2013.00867 24381551 PMC3865781

[B30] Moriguchi Y. Hiraki K. (2014). Behavioral and neural differences during two versions of cognitive shifting tasks in young children and adults. Dev. Psychobiol. 56, 761–769. 10.1002/dev.21145 23765326

[B31] Moriguchi Y. Minato T. Ishiguro H. Shinohara I. Itakura S. (2010). Cues that trigger social transmission of disinhibition in young children. J. Exp. Child Psychol. 107, 181–187. 10.1016/j.jecp.2010.04.018 20547394

[B32] Moriguchi Y. Sanefuji W. Itakura S. (2007). Disinhibition transmits from television to young children. Psychologia 50, 308–318. 10.2117/psysoc.2007.308

[B33] Moriguchi Y. Shinohara I. (2012). My neighbor: children’s perception of agency in interaction with an imaginary agent. PLoS ONE 7:e44463. 10.1371/journal.pone.0044463 22970225 PMC3436893

[B34] Morton J. B. Bosma R. Ansari D. (2009). Age-related changes in brain activation associated with dimensional shifts of attention: an fMRI study. Neuroimage 46, 249–256. 10.1016/j.neuroimage.2009.01.037 19457388

[B35] Motoshima Y. Shinohara I. Todo N. Moriguchi Y. (2014). Parental behaviour and children’s creation of imaginary companions: a longitudinal study. Eur. J. Dev. Psychol. 11, 716–727. 10.1080/17405629.2014.932278

[B36] Mulas F. Capilla A. Fernández S. Etchepareborda M. C. Campo P. Maestú F. (2006). Shifting-related brain magnetic activity in attention-deficit/hyperactivity disorder. Biol. Psychiatry 59, 373–379. 10.1016/j.biopsych.2005.06.031 16154541

[B37] Okamoto M. Dan H. Sakamoto K. Takeo K. Shimizu K. Kohno S. (2004). Three-dimensional probabilistic anatomical cranio-cerebral correlation via the international 10–20 system oriented for transcranial functional brain mapping. Neuroimage 21, 99–111. 10.1016/j.neuroimage.2003.08.026 14741647

[B38] Rossano F. Rakoczy H. Tomasello M. (2011). Young children’s understanding of violations of property rights. Cognition 121, 219–227. 10.1016/j.cognition.2011.06.007 21774921

[B39] Rueda M. R. Rothbart M. K. McCandliss B. D. Saccomanno L. Posner M. I. (2005). Training, maturation, and genetic influences on the development of executive attention. Proc. Natl. Acad. Sci. U.S.A. 102, 14931–14936. 10.1073/pnas.0506897102 16192352 PMC1253585

[B40] Sato H. Yahata N. Funane T. Takizawa R. Katura T. Atsumori H. (2013). A NIRS–fMRI investigation of prefrontal cortex activity during a working memory task. Neuroimage 83, 158–173. 10.1016/j.neuroimage.2013.06.043 23792984

[B41] Singer D. G. Singer J. L. (1990). The House of Make Believe. Cambridge, MA: Harvard University Press.

[B42] Strauss S. Ziv M. (2012). Teaching is a natural cognitive ability for humans. Mind Brain Educ. 6, 186–196.

[B43] Svendsen M. (1934). Children’s imaginary companions. Arch. Neurol. Psychiatry 32, 985–999.

[B44] Taylor M. (1999). Imaginary Companions and the Children Who Create Them. New York, NY: Oxford University Press.

[B45] Taylor M. Carlson S. M. Maring B. L. Gerow L. Charley C. M. (2004). The characteristics and correlates of fantasy in school-age children: imaginary companions, impersonation, and social understanding. Dev. Psychol. 40, 1173–1187. 10.1037/0012-1649.40.6.1173 15535765

[B46] Taylor M. Cartwright B. S. Carlson S. M. (1993). A developmental investigation of children’s imaginary companions. Dev. Psychol. 29, 276–285.

[B47] Thorell L. B. Lindqvist S. Nutley S. B. Bohlin G. Klingberg T. (2009). Training and transfer effects of executive functions in preschool children. Dev. Sci. 12, 106–113. 10.1111/j.1467-7687.2008.00745.x 19120418

[B48] Tomasello M. (2009). Why we Cooperate, Vol. 206. London: MIT press.

[B49] Tsujii T. Yamamoto E. Masuda S. Watanabe S. (2009). Longitudinal study of spatial working memory development in young children. Neuroreport 20, 759–763. 10.1097/WNR.0b013e32832aa975 19352206

[B50] Vaish A. Missana M. Tomasello M. (2011). Three-year-old children intervene in third party moral transgressions. Br. J. Dev. Psychol. 29, 124–130. 10.1348/026151010X532888 21288257

[B51] Vygotsky L. S. (1962). Thought and language. Cambridge, MA: MIT press.

[B52] Vygotsky L. S. (1967). Play and its role in the mental development of the child. Sov. Psychol. 5, 6–18.

[B53] Zelazo P. D. (2004). The development of conscious control in childhood. Trends Cogn. Sci. 8, 12–17. 10.1016/j.tics.2003.11.001 14697398

[B54] Zelazo P. D. (2006). The Dimensional Change Card Sort (DCCS): a method of assessing executive function in children. Nat. Protoc. 1, 297–301. 10.1038/nprot.2006.46 17406248

[B55] Zelazo P. D. Frye D. Rapus T. (1996). An age-related dissociation between knowing rules and using them. Cogn. Dev. 11, 37–63. 10.1016/S0885-2014(96)90027-1

[B56] Zelazo P. D. Muller U. Frye D. Marcovitch S. (2003). The development of executive function. Monogr. Soc. Res. Child Dev. 68, 1–27. 10.1111/j.0037-976x.2003.00261.x 14723273

[B57] Zhao H. Tanikawa Y. Gao F. Onodera Y. Sassaroli A. Tanaka K. (2002). Maps of optical differential pathlength factor of human adult forehead, somatosensory motor and occipital regions at multi-wavelengths in NIR. Phys. Med. Biol. 47, 2075–2093. 10.1088/0031-9155/47/12/306 12118602

